# Potential of Alpha-Mangostin-Loaded PLGA Nanoparticles for Cholangiocarcinoma Treatment

**DOI:** 10.3390/polym14204444

**Published:** 2022-10-20

**Authors:** Asma Tahir, Tullayakorn Plengsuriyakarn, Chuda Chittasupho, Kesara Na-Bangchang

**Affiliations:** 1 Center of Excellence in Pharmacology and Molecular Biology of Malaria and Cholangiocarcinoma, Chulabhorn International College of Medicine, Thammasat University (Rangsit Campus), Klongneung, Klongluang District, Pathum Thani 12121, Pathumthani, Thailand; 2 Department of Pharmaceutical Sciences, Faculty of Pharmacy, Chiang Mai University, Chiang Mai 50200, Thailand; 3 Drug Discovery and Development Center, Office of Advanced Science and Technology, Thammasat University (Rangsit Campus), Klongneung, Klongluang District, Pathum Thani 12121, Pathumthani, Thailand

**Keywords:** alpha-mangostin, PLGA, nanoparticles, cholangiocarcinoma, cell proliferation, apoptosis, cell invasion, cell migration

## Abstract

Alpha-mangostin (AM), a significant component isolated from the pericarp of mangosteen (*Garcinia mangostana* L.), has been demonstrated as a potential compound for the treatment of cholangiocarcinoma (CCA). Due to its hydrophobic nature, however, its clinical uses may be limited by its low aqueous solubility, poor stability, and low bioavailability. The present study aimed to formulate and characterize the AM-loaded PLGA nanoparticles (AM-PLGA-NPs) and further evaluate the antiproliferative and proapoptotic activities, including the inhibitory activities on CCA cell (CL-6 and HuCCT-1) invasion and migration. The AM-PLGA-NPs were prepared using PLGA MW 7000–17,000 and 38,000–54,000 by the solvent displacement method. The methods used to evaluate these activities included a MTT assay, flow-cytometry, QCM ECMatrix cell migration, and cell invasion assays, respectively. The optimized AM-PLGA-NPs were characterized for physical (particle size and morphology, polydispersity index, and zeta potential) and pharmaceutical (encapsulation efficiency, loading efficiency, and drug release profile) parameters. AM-PLGA-NPs showed relatively potent and selective antiproliferative and proapoptotic activities in both CCA cell lines in a concentration- and time-dependent manner. The results revealed that the PLGA nanoparticles could be a suitable nanocarrier to encapsulate AM for its delivery to the CCA cells.

## 1. Introduction

Cholangiocarcinoma (CCA) is an aggressive biliary tract malignancy with an increasing prevalence worldwide. The highest incidence is reported from the northeastern region of Thailand. The average incidence is 113 per 100,000 in men and 50 per 100,000 in women [1]. CCA is classified as intrahepatic, perihilar, or distal [2]. The major risk factors include a primary sclerosing cholangitis (PSC), a liver fluke infection (*Opisthorchis viverrini* and *Clonorchis sinensis*), and cirrhosis [3]. In Thailand, the primary risk factor is a *O. viverrini* infection caused by consuming raw or undercooked freshwater cyprinoid fish [3]. The conventional treatments for CCA are surgery, radiotherapy, and chemotherapy. Most patients present with an unrespectable or metastatic CCA and require treatment with chemotherapeutic drugs. The standard chemotherapeutic regimens are 5-fluorouracil (5-FU)-based or gemcitabine-based combination therapies with other drugs or targeted therapies, such as cisplatin or oxaliplatin [4]. However, the clinical efficacies of these drugs remain unsatisfactory.

Alpha-mangostin (AM) is a xanthone derivative isolated from the pericarp of mangosteen (*Garcinia mangostana* L.), which belongs to the Clusiaceae family, native to Southeast Asia [5]. AM possesses various biological and pharmacological activities, e.g., antioxidant, anti-inflammatory, antibacterial, and anticancer activities [6]. The anti-CCA activity of AM was investigated, in vitro, using the KKU-M214 cell line in a Ham-1 allograft hamster model [7]. The potent cytotoxic activity against the CCA cell proliferation was demonstrated with an IC_50_ of 1.36 g/mL, with no significant cytotoxic effects on the normal white blood cells. The antiproliferative effect was through the apoptosis induction via the mitochondrial pathway. In addition, a marked reduction in the tumor size was shown with a comparative potency to the standard drug gemcitabine. AM was also shown to exert anticancer activities against hepatocellular carcinoma [8], bone, tongue, and breast cancers [9], as well as colon, lung, and gastric cancers [10]. It was also shown to suppress the viability and the epithelial-mesenchymal transition of the pancreatic cancer cells by downregulating the PI3K/AKT pathway [11]. The limitations of using AM in various types of cancers are the low aqueous solubility and the poor target selectivity toward the tumor cells. The strategies to overcome these limitations would support the chemotherapeutic uses of AM, including in CCA.

The encapsulation of the hydrophobic drugs into the nanoparticulate systems has been applied as a drug delivery system for both chemotherapeutic drugs [12] and traditional medicines [13], to improve their systemic bioavailability, selectivity to tumor cells, and thus, clinical effectiveness [14,15,16,17]. Poly (lactic-co-glycolic acid) or PLGA is a biocompatible and biodegradable polymer approved by the US FDA for clinical use [18]. It is used for the preparation of polymeric nanoparticles and is also widely used as a drug delivery system for chemotherapeutic drugs and traditional medicines. Examples include docetaxel-loaded PLGA-PEG [19] and curcumin-loaded PLGA [20] nanoparticles, which enhance the water-solubility and anticancer activities of both compounds. To our knowledge, there has been no study that examines the delivery of AM via nanoparticles in CCA. The present study aimed to formulate and characterize the AM-loaded PLGA nanoparticles (AM-PLGA-NPs) and further evaluated their antiproliferative and proapoptotic activities, including the inhibitory activities on the CCA cell invasion and migration.

## 2. Materials and Methods

### 2.1. Chemicals

AM and 5-FU were purchased from Wako Pure Chemical Industries, Ltd., Osaka, Japan. PLGA (50:50) with the molecular weights of 7000–17,000 (Resomer^®^502) and 38,000–54,000 (Resomer^®^504), the MTT reagent (3-[4,5-dimethylthiazol-2yl]-2,5-diphenyl-tetrazolium bromide) was purchased from Sigma-Aldrich (St. Louis, MO, USA). Acetone and DMSO were purchased from Fisher Scientific, Co. (Pittsburgh, PA, USA). Poloxamer 407 was purchased from BASF (Florham Park, NJ, USA). The CL-6 cell line was kindly provided by Associate Professor Dr. Adisak Wongkajornsilp, Department of Pharmacology, Faculty of Medicine (Siriraj Hospital), Mahidol University. The normal fibroblast cell line, OUMS-36T-1F, was purchased from the JCRB Cell Bank (Japanese Collection of Research Bioresources Cell Bank, Osaka, Japan). RPMI (Roswell Park Memorial Institute medium) and DMEM (Dulbecco’s Modified Eagle Medium), fetal bovine serum (FBS), antibiotic-antimycotic solution, and FITC Annexin V/Dead cell apoptosis KIT with FITC annexin V and propidium iodide (PI) were purchased from Life Technologies (Carlsbad, CA, USA). Phosphate buffer saline (PBS) and dimethylsulfoxide (DMSO) were purchased from Ameresco (Cincinnati, OH, USA).

### 2.2. Preparation of the Alpha-Mangostin-Loaded PLGA Nanoparticles

AM-PLGA-NPs were prepared using the solvent displacement method (nanoprecipitation method) with a slight modification [21]. AM (2 mg) and PLGA (50 mg) were dissolved in acetone and added dropwise (drop rate: 10 mL/h) to 15 mL of 0.1% poloxamer 407 under stirring (550 rpm), using a syringe pump (KD Scientific, Holliston, MA, USA). The suspension was continuously stirred for 15 min to remove the organic solvent. 

### 2.3. Characterization of the Particle Size, Distribution, and Zeta Potential Parameters

The prepared AM-PLGA-NPs were characterized for the average particle size, polydispersity index (PDI), and the zeta potential by dynamic light scattering (DLS) (Zeta sizer, Malvern Instrument Ltd., Worcestershire, UK) [21]. To measure the nanoparticle size, charge, and polydispersity index, 1 mL of freshly prepared AM-PLGA NPs or a PLGA blank was added to a microcentrifuge tube and centrifuged at 13,000× *g* for 10 min (4 °C). The supernatant was discarded, and the pellets were resuspended in 1 mL of deionized water. The experiment was repeated three times.

### 2.4. Morphology Examination

The morphology of the AM-PLGA-NPs and PLGA-NPs were examined under a transmission electron microscope (TEM). A few microliters of the NP suspension was placed on a carbon-coated film, 300 mesh copper grid, and was allowed to dry. The analysis was carried out by digital micrograph and a soft imaging viewer software to capture and analyze all of the images.

### 2.5. Determination of the Encapsulation Efficiency (%EE) and the Loading Efficiency (%LE)

The freshly prepared AM-PLGA-NP suspension (1 mL) was added to a microcentrifuge tube and centrifuged at 13,000× *g* for 10 min (4 °C). The supernatant was discarded, and 1 mL of DMSO was added to dissolve the drug and polymer. The mixture was sonicated until fully dissolved. The concentration of AM was measured at the maximum wavelength of 322 nm using a microplate reader. The encapsulation efficiency (%EE) and loading efficiency (%LE) of AM-PLGA-NPs were calculated as follows:

(1) $$\mathrm{Encapsulation}\;\mathrm{Efficiency}\;\left(\%\mathrm{EE}\right)=\frac{\mathrm{Amount}\;\mathrm{of}\;\mathrm{alphamangostin}\;\mathrm{encapsulated}\;\mathrm{in}\;\mathrm{PLGA}\;}{\mathrm{Total}\;\mathrm{amount}\;\mathrm{of}\;\mathrm{alpha}\;\mathrm{mangostin}\;}\times 100\%$$

(2) $$\mathrm{Loading}\;\mathrm{Efficiency}\;\left(\%\mathrm{DL}\right)=\;\frac{\mathrm{Amount}\;\mathrm{of}\;\mathrm{alphamangostin}\;\mathrm{encapsulated}\;\mathrm{in}\;\mathrm{PLGA}\;}{\mathrm{Total}\;\mathrm{amount}\;\mathrm{of}\;\mathrm{PLGA}\;}\times 100\%$$

### 2.6. In Vitro Drug Release Study

AM-PLGA-NPs (1 mL) was centrifuged at 13,000× *g* for 10 min (4 °C). The supernatant was discarded, and 1 mL of PBS was added to dissolve the drug and polymer. The mixture was redispersed by sonication, thoroughly mixed, and placed in an incubator shaker (37 °C, 110 rpm) for 7 days to simulate the peristaltic conditions. Next, 100 µL aliquots were collected at 1, 3, and 6 h, and at 1, 2, 3, 4, 5, 6, and 7 days. The amount of AM released into the medium was measured using UV spectrophotometry at 322 nm, according to the formula:

(3) $$\mathrm{Cumulative}\;\mathrm{release}\;\mathrm{of}\;\mathrm{alpha}-\mathrm{mangostin}\;\left(\%\right)=\left(\frac{\mathrm{DL}-\mathrm{DR}}{\mathrm{DL}}\right)\times 100$$

where DL is the amount of drug-loaded in the NPs. DR is the amount of drug that remains in the NPs.

### 2.7. Antiproliferative and the Proapoptotic Activities, and the Inhibitory Activities on Cell Migration and Invasion

#### 2.7.1. Cell Culture

The human CCA cell lines, CL-6 and HUCCT-1, as well as the normal fibroblast cell line OUMS-36T-1F, were used in this study. All CCA cell lines were cultured in a RPMI 1640 medium. The OUMS-36T-1F cell line was cultured in DMEM. Both culture media were prepared as complete media with a supplement of 10% (*v*/*v*) heated FBS and 1% 100 IU/mL antibiotic-antimycotic solution. The cultures were maintained at 37 °C under 5% CO_2_ atmosphere and 95% humidity (HERRACELL 150i, Thermo Scientific, Waltham, MA, USA). The cells were preserved in 10% DMSO supplemented with 90% FBS and stored in a liquid nitrogen tank (−180 °C).

#### 2.7.2. Preparation of the Test Materials

The AM-PLGA-NPs and the blank NPs were dissolved in 1 mL of deionized water. AM and 5-FU were dissolved in 50% ethanol. All test materials were serially diluted (1:2) with a complete media in order to obtain the working solution at eight final concentrations.

#### 2.7.3. Antiproliferation Assay

The standard MTT assay was used to determine the cell viability. Briefly, 8000 cells (CL-6, HuCCT-1, or OUMS-36T-I1) were seeded onto each well of a 96-well plate and incubated at 37 °C in 5% CO_2_ for 24 h. Following the incubation, the cells were treated with AM-PLGA-NPs, free AM, and 5-FU (positive control) at different concentrations (125–0.97 μg/mL) and incubated at 37 °C in 5% CO_2_ for 48 and 72 h. The MTT reagent (0.5 mg/mL) was added to each well and further incubated for an additional 3.5 h. The medium was removed, and the cells were lysed with 100 μL of DMSO and incubated at room temperature (25 °C) for 15 min. The absorbance was measured at 570 nm using a UV visible spectrophotometer (Vario scan flash, Thermo fisher scientific, Waltham, MA, USA). The cell viability (%) and IC_50_ were calculated using Calcusyn^®^ version 1.1 (Biosoft, London, UK). All experiments were performed in triplicate. The cell viability and selectivity index (SI) were calculated using the following equations:

(4) $$\mathrm{Cell}\;\mathrm{viability}\;\left(\%\right)=\left(\frac{\mathrm{Absorbance}\;\mathrm{of}\;\mathrm{cell}\;\mathrm{treated}\;\mathrm{with}\;\mathrm{samples}}{\mathrm{Absorbance}\;\mathrm{of}\;\mathrm{negative}\;\mathrm{control}}\right)\times 100$$

(5) $$\mathrm{Selectivity}\;\mathrm{index}\;\left(\mathrm{SI}\right)=\frac{\mathrm{IC}50\;\mathrm{of}\;\mathrm{normal}\;\mathrm{cells}}{\mathrm{IC}50\;\mathrm{of}\;\mathrm{cancer}\;\mathrm{cells}}$$

#### 2.7.4. Apoptosis Assay

The proapoptotic activities of free AM, AM-PLGA-NPs, blank NPs, and 5-FU (reference control) were evaluated using an Annexin V-FITC assay kit (Sigma-Aldrich, St. Louis, MO, USA), according to the manufacturer’s protocol. Briefly, all three cell lines (CL-6, HuCCT-1 and OUMS-36T-1F) were seeded onto 25 mL culture flasks (400,000 cells) and incubated for 24 h. The cells were treated with all test materials at IC_25_ and IC_50_ for 48 and 72 h at 37 °C in 5% CO_2_. The cells were harvested with trypsin and centrifuged at 1500–2300× *g* for 5 min (25 °C). The cells were washed twice with PBS to remove any remaining media and stained with 5 μL of Annexin V-FITC and propidium iodide. The cell analysis was performed by flow cytometry.

#### 2.7.5. Cell Migration Assay

The effects of all of the test materials (free AM, AM-PLGA-NPs, blank NPs, and 5-FU) on the cell migration were determined using a QCM ECMatrix cell migration chamber 96-wells (Millipore, Burlington, MA, USA), according to the manufacturer’s protocol with modification. In brief, both CCA cell lines, CL-6, HuCCT-1 and OUMS-36T-1F, were pretreated with each test material at IC_25_ and IC_50_ for 48 and 72 h. The prewarmed serum-free medium (150 µL) was added to the lower chamber of the migration plate, and the cells were gently resuspended in a 100 μL medium without chemoattractant and added to the migration chamber and incubated at 37 °C under 5% CO_2_ for 24 h. The cells and culture medium were gently discarded from the top side of the inserts. The migration chamber plate was placed onto the new feeder tray, containing 150 μL of the prewarmed cell detachment solution and incubated at 37 °C for 30 min. The migration chamber plate was gently tilted back and forth during the incubation to completely remove the cells. The lysis buffer/dye solution (50 μL) was added to each well of the feeder tray containing the cell detachment solution (150 μL total volume). Following the incubation (25 °C, 15 min), the mixture was transferred to a new 96-well plate, and the absorbance was measured at 480/520 nm. The experiment was repeated three times. 

#### 2.7.6. Cell Invasion Assay

The effects of all of the test materials (free AM, AM-PLGA-NPs, blank NPs, and 5-FU) on the cell invasion were determined using a QCM ECMatrix cell invasion chamber 96-wells (Millipore, MA, USA), according to the manufacturer’s protocol with modification. In brief, all three cell lines were pretreated with each test material at IC_25_ and IC_50_ for 48 and 72 h. The prewarmed serum-free medium (100 µL) was added to the inserts of the invasion assay plate coated with an extracellular matrix layer and rehydrated at 25 °C for 1–2 h. The medium from the inserts was carefully removed, and 150 µL of the medium containing 10% FBS was added to each well of the feeder tray. The cells (8000–10,000 cells per 100 μL) were added to the invasion chamber and then incubated at 37 °C under 5% CO_2_ for 24 hr. The cells and the culture medium were gently discarded from the top side of the inserts. The inserts were rinsed by placing the chamber plate onto a new 96-well feeder tray containing 150 µL of PBS and incubated at 25 °C for 1 min. The invasion chamber plate was placed back in the 96-well feeder tray containing 150 μL of the prewarmed cell detachment solution and was incubated at 37 °C under 5% CO_2_ for 30 min. The lysis buffer/dye solution (50 μL) was added to each well of the feeder tray containing the cell detachment solution. Following the incubation (25 °C, 15 min), the mixture was transferred to a new 96-well plate, and the absorbance was measured at 480/520 nm. The experiment was repeated three times.

### 2.8. Statistical Analysis

The statistical analyses were performed using SPSS version 21.0 (SPSS Inc., Armonk, NY, USA). The data are presented as median and range values. The difference between the two groups of quantitative variables was determined using the Mann–Whitney U test. The statistical significance level was set at α = 0.05.

## 3. Results

### 3.1. Preparation and Characterization of the AM-PLGA-NPs

**Figure 1:**
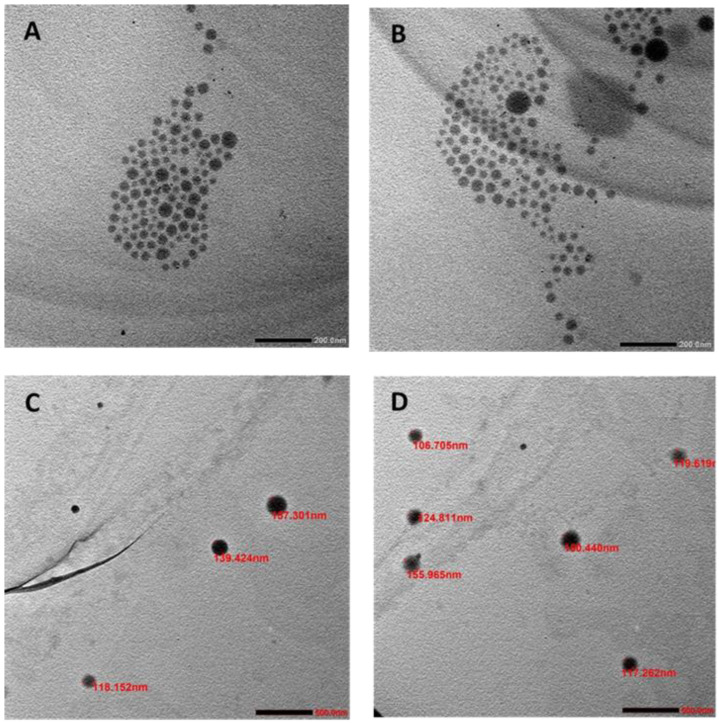
TEM images of (**A**) AM-PLGA-NPs (**B**) PLGA-NPs (**C**) large-scale of the AM-PLGA-NPs, and (**D**) large-scale of PLGA-NPs.

The nanoprecipitation method successfully produced alpha-mangostin encapsulated in the PLGA nanoparticles (AM-PLGA-NPs). The AM-PLGA-NPs and blank PLGA-NPs showed suitable physical (particle size, PDI, and zeta potential) and pharmaceutical (%EE and %LE) properties (Table 1). The TEM images confirmed the particle size to be within the range of 118.9–221 nm for AM-PLGA-NPs and the blank PLGA-NPs (Figure 1A,B). Both NPs showed a spherical shape with a smooth surface, and monodispersion without aggregation.

### 3.2. In Vitro Release of the AM-PLGA-NPs

**Figure 2:**
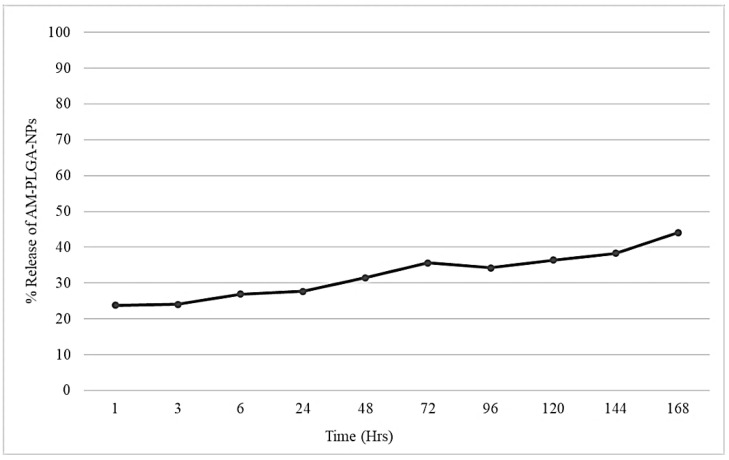
In vitro release profile of the AM-PLGA-NPs during 168 h period (7 days).

The cumulative release of AM from the AM-PLGA-NPs in the simulated physiological conditions (PBS pH 7.4) over a one-week period, is shown in Figure 2. The release profile was biphasic with an initial rapid release of 27.71% at 24 h, followed by a slow release of 44.1% at 168 h (7 days).

### 3.3. Antiproliferative Activity

The antiproliferative activities of the test materials against the CCA cell lines, CL-6 and HuCCT-1, and the normal cell line OUMS-36T-1F, are summarized in Table 2. At 48 h of exposure, the antiproliferative activities of the AM-PLGA-NPs against both CCA cell lines were significantly higher than free AM and 5-FU. For the CL-6 cells, the potencies of the activity were 1.69- and 3.4-fold higher than free AM and 5-FU, respectively. The corresponding potencies against HuCCT-1 were 1.08- and 3.23-fold higher, respectively. The AM-PLGA-NPs were more selective to the CCA cells than free AM was, but the NPs were less selective than 5-FU. The selectivity index (SI) values of the AM-PLGA-NPs, free AM, and 5-FU for the CL-6 cells were 3.0, 1.0, and 5.6, respectively. The corresponding SI values for HuCCT-1 were 2.3, 1.1, and 5.3, respectively (Table 2). At 72 h of exposure, the antiproliferative activity of the AM-PLGA-NPs had significantly increased in both cell lines. The antiproliferative potency of the AM-PLGA-NPs against both CCA cell lines was generally significantly higher than free AM and 5-FU. For the CL-6 cells, the potencies of the activity were 3.2- and 8.41-fold that of free AM and 5-FU, respectively. The corresponding potencies against HuCCT-1 were 4.47- and 0.82-fold, respectively. The AM-PLGA-NPs were more selective to the CCA cells than free AM, but less selective than 5-FU. The SI values of the AM-PLGA-NPs, free AM, and 5-FU for the CL-6 cells were 4.0, 1.0, and 3.6, respectively. The corresponding SI values for HuCCT-1 were 5.2, 0.76, and 41.0, respectively (Table 2).

### 3.4. Apoptotic Activity

**Figure 3:**
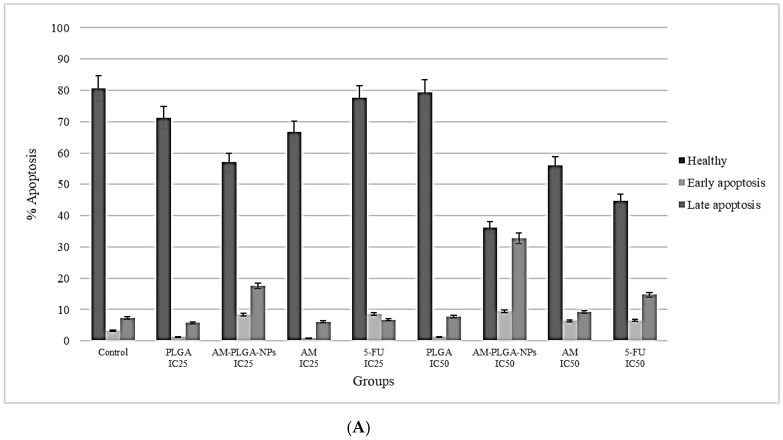

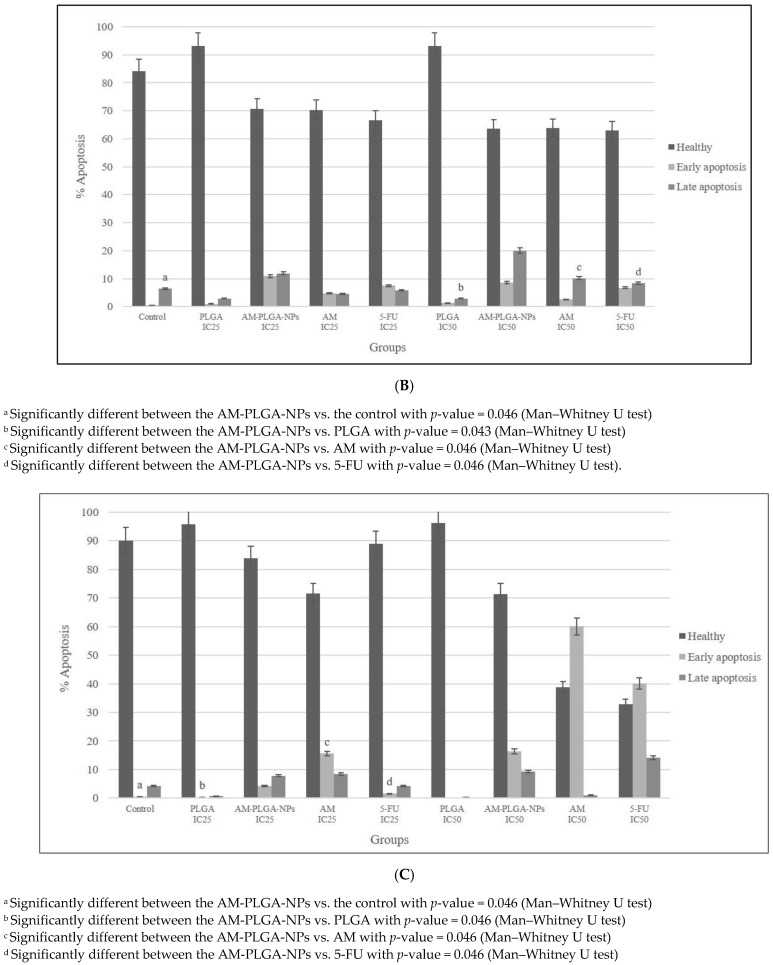
Induction of **the** apoptosis in the human CCA cell lines (**A**) HuCCT-1, (**B**) CL-6, and (**C**) normal cell line OUMS-36T-1F in the untreated cells, and the cells exposed to free AM, AM-PLGA-NPs, blank PLGA nanoparticles (PLGA), and 5-FU at IC_25_ and IC_50_ concentrations. Data presented as median (range) of the triplicate experiments.

The exposure of the HuCCT-1 and CL-6 cells to the AM-PLGA-NPs resulted in a significant decrease in the population of healthy cells, while increasing the populations of the late and early apoptotic cells, compared with the control and blank PLGA-NPs (Figure 3).

The proapoptotic activity of the AM-PLGA-NPs at the IC_25_ and IC_50_ concentrations in HuCCT-1 and CL-6 showed different patterns. In HuCCT-1, the inducing activities on the early-stage apoptosis of the AM-PLGA-NPs at both concentrations were similar (3.81 and 3.85-fold of the control, respectively). The inducing activities in CL-6 were also similar (11.32- and 10.99-fold of the control, respectively). The inducing activity on the late stage apoptosis in HuCCT-1 was, however, higher than CL-6 following the exposure to the AM-PLGA-NPs at the IC_25_ concentration. A significant increase in the apoptosis induction at the late stage was observed, following the exposure of CL-6 to the AM-PLGA-NPs, compared with the untreated control, free AM, PLGA, and 5-FU. In the normal cells (OUMS-36T-1F), a significant increase in the apoptosis induction at the early stage was observed following the exposure of the cells to the AM-PLGA-NPs, when compared with the untreated control, free AM, PLGA, and 5-FU.

### 3.5. Inhibitory Activities on the Cell Migration and Invasion 

**Figure 4:**
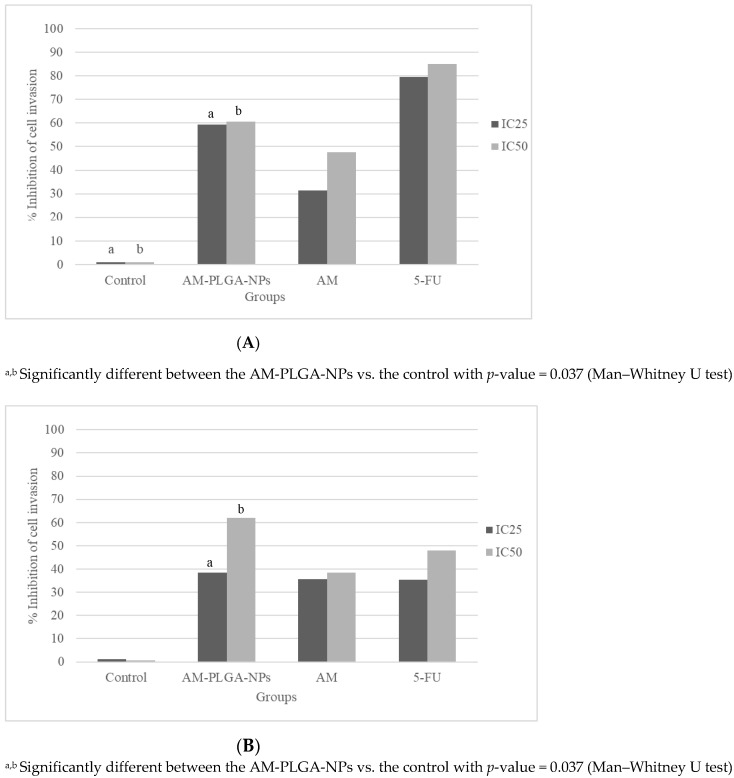

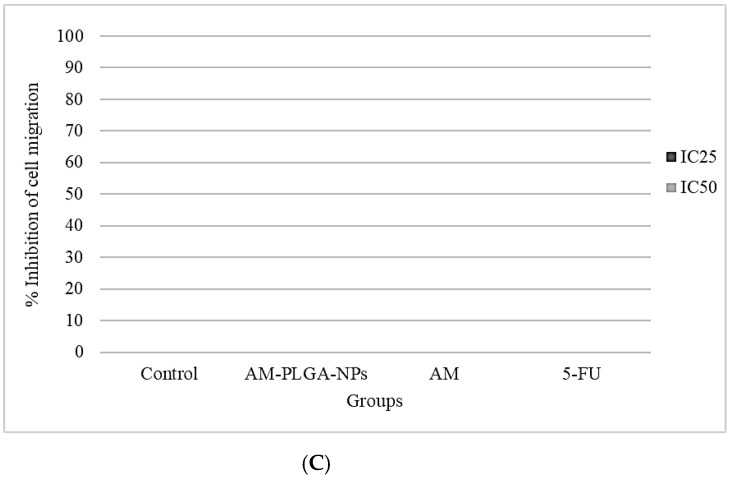
Inhibitory effects on the migration of (**A**) HuCCT-1, (**B**) CL-6, and (**C**) OUMS-36T-1F cell lines following the exposure to the AM-PLGA-NPs, blank PLGA, free AM, and 5-FU for 72 h. Data presented as median (range) of the triplicate experiments.

**Figure 5:**
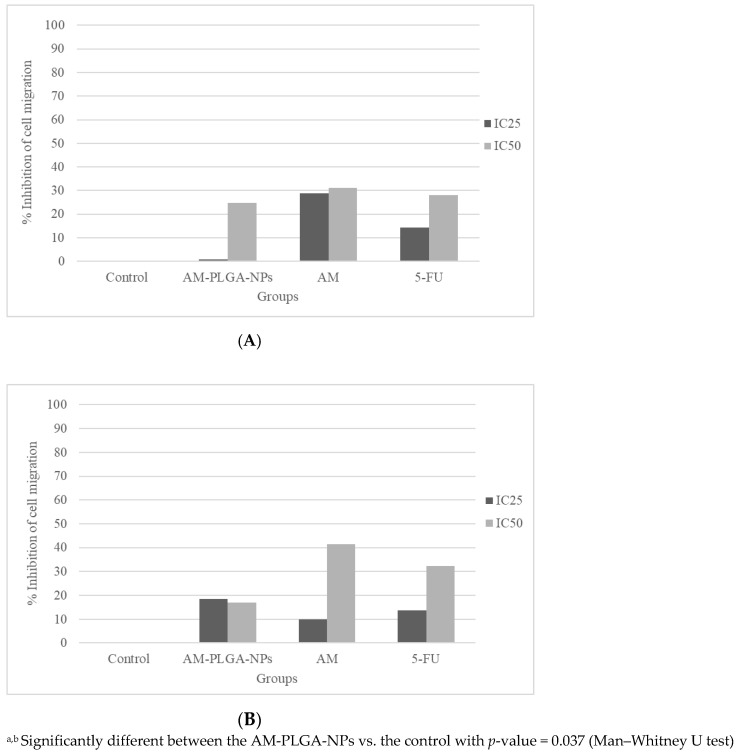

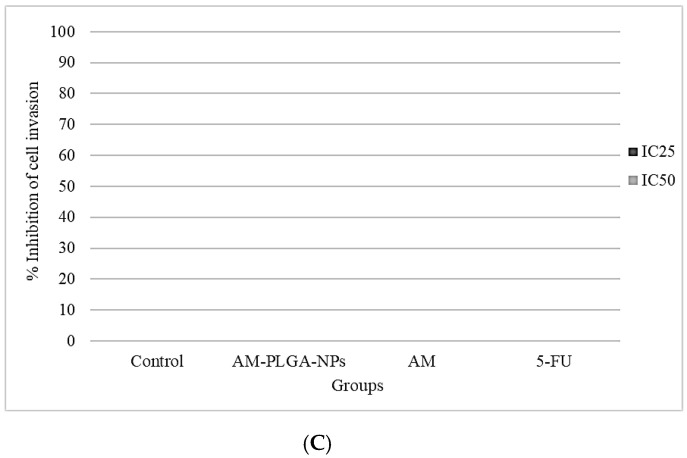
Inhibitory effects on the invasion of (**A**) HuCCT-1, (**B**) CL-6 and (**C**) OUMS-36T-1F cell lines following the exposure to the AM-PLGA-NPs, blank PLGA, free AM, and 5-FU for 72-h. Data presented as median (range) of the triplicate experiments.

The inhibitory activities of the AM-PLGA-NPs on the migration and invasion of both CCA cell lines were concentration- and time-dependent, while no inhibitory effect was found with the OUMS-36T-1F cells (Figure 4 and Figure 5).

The inhibitory activity on the HuCCT-1 cell migration of the AM-PLGA-NPs at the IC_50_ concentration was significantly higher than in the untreated control. In the CL-6 cells, a significant inhibition was observed at both IC_25_ and IC_50_ concentrations. No inhibitory activity was observed in the OUMS-36T-1F cell line (Figure 5). The inhibitory effect was lower than 5-FU and the PLGA-NPs in both the CL-6 (16.9% vs. 32.3.9% vs. 41.5%), and HuCCT-1 (24.8% vs. 28.0% vs. 31.2%) cells (Figure 4).

The inhibitory activity of the AM-PLGA-NPs on the cell invasion at both IC_25_ and IC_50_ concentrations was significantly higher than in the untreated control in both the HuCCT-1 and CL-6 cell lines. No inhibitory activity was found in the OUMS-36T-1F cell line (Figure 5). The inhibitory activity of the AM-PLGA-NPs on the CL-6 cell invasion was higher than 5-FU (61.9% vs. 47.9%), while the inhibitory effect of 5-FU was higher in the HuCCT-1 cell line (60.5% vs. 85.1%) (Figure 4).

## 4. Discussion

The main objective of this study was to formulate and characterize the PLGA nanoparticles encapsulating alpha-mangostin in order to improve the bioavailability of AM, and thus, the anti-CCA activity [2]. The AM-PLGA-NPs with optimized physical and pharmaceutical properties were successfully produced using the nanoprecipitation method. The advantages of PLGA over other polymers include its low toxicity, controlled and sustained release properties, and its enhanced permeability and retention (EPR) effect [22]. The PLGA-NPs were prepared using the solvent displacement method, as previously reported by Muhamad et al. (2020) [21]. However, the factors affecting the successful NP preparation were different, including the concentration of the encapsulated drug (alpha-mangostin and atractylodin) and the concentration of the surfactant. In addition, we have also optimized the formulation to obtain the highest %EE and %LE, as shown in Table 1.

The particle size plays a pivotal role in the biological activity of the NPs. The size of the PLGA-NPs was influenced by the solubility of the drug and the polymer in the organic solvent and the stability parameters used (temperature and incubation time). The solvent used to prepare the PLGA-NPs should be water-miscible and easily removed through evaporation, extraction, or both [23]. The common solvents for nanoprecipitation are acetone, acetonitrile, dimethylacetamide, dimethylformamide, dimethylsulfoxide, 2-pyrrolidone, *N*-methyl-2-pyrrolidone, polyethylene glycol, and tetrahydrofuran [24]. Acetone is one of the most popular solvents among them. Temperature markedly influences the stability of the PLGA-NPs. However, according to our experience with the PLGA-NP storage, the size of the PLGA-NPs did not change when stored at a low temperature, e.g., 4 °C [25,26]. Time also affects the particle size, depending on the surface charge, the storage temperature, and the medium used to incubate the nanoparticles [27]. The lower the temperature, the thinner the AM-particles and the larger the surface area. The longer the incubation time, the smaller the particle size and the more extensive the size distribution. All of the optimized PLGA-NPs had an optimal particle size below 235 nm (acceptable size = 50–500 nm) and a PDI of 0.189 (acceptable PDI = 0.1–0.3). These properties promote the cellular uptake by endocytosis and the homogenous size distribution within the NPs. Furthermore, the zeta potential (surface charge of the NPs in the disperse medium) was less than −32 mV. The NPs with a zeta potential of less than −30 mV, or more than +30 mV are considered highly stable and are able to redisperse easily without aggregation. Based on the DLVO electrostatic theory [28], the stability of the emulsion and the colloids depends on the net surface charge, which is the balance between the attractive van der Waals’ forces and the electrical repulsion. If the zeta potential falls below a certain level, the emulsion droplets or colloids will aggregate due to the attractive forces. Conversely, a large zeta potential (either positive or negative), typically greater than ±30 mV, maintains a stable system. The zeta potential charges also influence the tumor cell penetration, and the negatively charged NPs have a prolonged blood circulation time. In previous studies, the mean diameters of the docetaxel-PEG-PLGA-NPs and the paclitaxel-PLGA-NPs were found to be relatively smaller, i.e., 186–267 nm [29] and <200 nm [30], respectively. This difference could be due to difference in the concentrations of the polymers or the organic solvents, and the method used to synthesize the nano-formulation. However, the prepared AM-PLGA-NPs had comparable properties with the curcumin and gemcitabine co-loaded polymeric NPs prepared by the double emulsion solvent evaporation (particle size, PDI, and negative zeta potential of 236 ± 0.094 nm, 0.218 ± 0.037, and −19.7 ± 6.91 mV, respectively) [31], and the topotecan-loaded PLGA NPs (243.2 ± 4 nm, 0.107 ± 0.01 and −2.36 ± 0.6 mV, respectively) [32]. Altogether, these results suggest that the PLGA-NPs encapsulating hydrophobic drugs produce nano-formulations with a small particle size and a homogeneous particle distribution without aggregation or cohesion, which influences the stability of the nanoparticles in the suspension. The examination of the particle size and morphology of the AM-PLGA-NPs using a TEM revealed a smooth spherical shape with an average particle size ranging from 118.9 to 221 nm. The diameter measured with the TEM was smaller than the DLS, as the TEM analysis was performed in dry form or on air-dried NPs with a neutral environment, while DLS analysis was performed in a hydrodynamic state with the core and surrounding charge layer.

Previous studies reported that PLGA could encapsulate AM to produce NPs. Various parameters were optimized, including the concentrations of the drug/compound, the polymer, as well as the surfactant, and the volume of the organic solvent to achieve the maximum %EE and %LE. A low concentration of the drug/compound and a high concentration of PLGA resulted in a high %EE [33]. The best-optimized drug:polymer ratio to obtain the highest %EE and %LE was found to be 1:25 (e.g., 2 mg of drug and 50 mg of polymer). The surfactant or stabilizer enhances the wettability and dispersibility of the drug and limits the recrystallization of the drug to prevent precipitation, by absorbing the outer layer of the encapsulated drug particles. The concentration of the surfactant affects these properties. Too low of a concentration causes the aggregation of the drug particles, while too high of a concentration reduces the drug incorporation due to the interaction between the drug and stabilizer. The concentration of the stabilizer (poloxamer 407) also significantly affects the %EE and %LE. Decreasing the surfactant concentration from 1% to 0.1% increased the %EE from 30% to 64% (2.1-fold) and the %LE from 1.08% to 2.5% (2.3-fold). The volume of the organic solvent had no significant effect on the %EE and %LE. In this study, the drug-releasing profile of the AM-PLGA-NPs was biphasic, with an initial fast release during the first 24 h, followed by a sustained release thereafter. This suggests that AM was either entrapped on the surface of the PLGA NPs or loaded inside the core of PLGA. The initial fast release phase is attributed to the entrapment on the surface of the polymer. This results from the weak bonding between the drug and the polymer, and thus, the drug diffuses out very quickly. The sustained released phase could be attributed to either the diffusion of the drug localized in the PLGA nanoparticle core, or the degradation of the polymer matrix over time. The mechanism of the AM release from the PLGA-NPs could be via a passive diffusion, erosion, or polymer degradation. Notably, AM was not released completely during the observation period (maximum release 44.1%). Due to the polymer entanglement and the interaction between PLGA and AM, the encapsulated AM may take longer than a month to be released from the polymer by degradation. The FTIR spectra indicated the hydroxyl groups of the AM hydrogen bonding with the carbonyl group of PLGA [34]. The antiproliferative activities of the AM-PLGA-NPs against the two CCA cell lines, CL-6 and HuCCT-1, were concentration- and time-dependent and more selective to the CCA cell lines than the normal cell line (SI 4.0–5.2). The potencies of the activity were generally higher than 5-FU (3–6-fold) and free AM (0.5–3-fold). No antiproliferative activity of the blank PLGA-NPs was observed in any cell line [21]. These results indicate that the AM-PLGA-NPs and free AM exerted antiproliferative activities on both CCA cell lines, but the activity was significantly enhanced when AM was loaded in the PLGA-NPs. The in vitro release profile of the AM-PLGA-NPs correlated well with its cytotoxic (antiproliferative) activity against the CCA cells. The initial fast release phase resulted in an immediate cytotoxic effect, while the sustained release phase resulted in a prolonged cytotoxic effect. The antiproliferative activity of AM has previously been demonstrated in the human M214 CCA cell line [7]. The PLGA-NPs have been shown to enhance the antiproliferative activity of the encapsulated chemotherapeutic drugs, such as topotecan, paclitaxel, and gemcitabine [32,35,36]. Based on the SI, the AM-PLGA-NPs appear to be very selective towards both CCA cell lines compared to the normal cell line. The blank PLGA nanoparticles did not produce any cytotoxic effect against the CCA cells. The particle size is an essential factor that influences the physical and pharmaceutical properties of the AM-PLGA-NPs and the biological activity. Although the mechanism of the cellular uptake of the AM-PLGA-NPs was not investigated in this study, the cellular uptake by endocytosis has been reported with the PLGA-NPs of a 236 nm particle size [37]. In a previous study, the macropinocytic, caveolae- and clathrin-mediated endocytic pathways have also been reported to be involved in the uptake of PLGA, regardless of the particle size (230 or 160 nm) [38].

Apoptosis is a highly regulated and organized cell death process that prevents and controls the development of cancer by removing the unwanted cells through the production of various enzymes. The crude extract of *G. mangostana* was shown to induce M214 CCA cell apoptosis through the mitochondrial pathway by inducing caspase-3, p53, Bax, and Bcl-2 protein expressions, as well as by reducing the cell proliferation through cell-cycle arrest at the G_1_ phase [7]. In this study, the proapoptotic activity of free AM, AM-PLGA-NPs, blank PLGA-NPs, and 5-FU on CL-6, HuCCT-1 CCA, and OUMS-36T-1F cell lines were observed during the 48- and 72-h exposure periods. The proapoptotic activity of the AM-PLGA-NPs against both CCA cell lines was concentration- and time-dependent and also stage- and CCA cell type-specific. Free AM, AM-PLGA-NPs, and 5-FU induced apoptosis in both CCA cell lines, while the blank PLGA-NPs did not. The AM-PLGA-NPs significantly induced early and late apoptosis in both CCA cells, compared with free AM and 5-FU. In agreement with this observation, the apoptotic activity of the AM-PLGA-NPs was reported in colorectal cancer [39].

The cancer cell migration and invasion are critical components of cancer cell metastasis, which is the major cause of mortality and death in cancer patients. The antimetastatic activity of AM was demonstrated in various cancer stem cells, such as PC-3 human prostate carcinoma cells. The underlying molecular mechanism is by inhibition of the expression of matrix metalloproteinase-2/9 and urokinase-plasminogen through the JNK signaling pathway [40]. AM was shown to suppress the subsistence of lung cancer cells and exerted an antimetastatic activity by inhibiting the cell migration and invasion and decreasing the actin cytoskeleton of cancer cells [41]. Encapsulating chemotherapeutic agents or herbal medicines in the NPs is an attractive strategy to improve their antimetastatic properties. The co-delivery of the hydroxyethyl starch-polylactide (HES-PLA)-NPs, doxorubicin and TGF-β receptor inhibitor LY2157299 was shown to suppress the primary tumor and the distant metastasis in breast cancer 4T1 cells [42]. The doxorubicin and cisplatin co-loaded NPs were also shown to exert a higher antitumor efficiency for metastatic lung cancer, compared with free doxorubicin and cisplatin [43]. The present study demonstrated the inhibitory activities of the AM-PLGA-NPs on the CCA cell migration and invasion, as well as the selectivity to only CCA cells in a concentration- and time-dependent manner. No inhibitory effects of the AM-PLGA-NPs, free AM, blank PLGA-NPs, or 5-FU were observed in the normal cell line (OUMS-36T-1F). The PLGA-NPs did not exhibit any inhibitory activity in any cells. The potencies of the activity on the cell migration and invasion of the AM-PLGA-NPs in both CCA cell lines were generally about 1.1–1.9-fold that of 5-FU. However, it should be noted that the AM-PLGA-NPs displayed a higher potency of the inhibitory activity on the HuCCT-1 cell invasion.

## 5. Conclusions

The AM-PLGA-NPs were successfully prepared as a drug delivery system to enhance the aqueous solubility, thus improving the anti-CCA activities of AM. The optimized AM-PLGA-NPs with higher encapsulation and loading efficiencies provided superior antiproliferative and apoptosis-inducing activities, as well as a greater inhibitory activity on the CCA cell migration and invasion compared to free AM. The PLGA-NPs could be a potential drug delivery system for AM in CCA. The underlying molecular mechanisms of the AM-PLGA-NPs involved in these activities should be further investigated. 

## Figures and Tables

**Table 1:** Physical and pharmaceutical properties (particle size, PDI, zeta potential, %EE, and %LE) of the blank PLGA-NPs and the AM-PLGA-NPs.

Formulation	Particle Size (nm)	PDI	Zeta Potential (mV)	EE (%)	LE (%)
Blank PLGA NPs	226.9 (226.4–227.9)	0.179 (0.155–0.189)	−34.9 (−34.8–35.4)	-	-
AM-PLGA	230.1 (229.3–235.7)	0.122 (0.099–0.132)	−32.1 (−32.1–32.8)	64 (59–67)	2.5 (2.3–2.68)

Data are presented as median (range) values.

**Table 2:** Antiproliferative activities (IC_50_) and the SI of free AM, AM-PLGA-NPs, and 5-FU on the CL-6, HuCCT-1, and OUMS-36T-1F cell lines after 48 h of exposure. Data presented as median (range) of triplicate experiments.

Cell lines	Test Compounds	IC_50_ (µg/mL) 48 h		IC_50_ (µg/mL) 72 h	
		Median (Range)	SI	Median (Range)	SI
CL6	AM	11.70 (9.70–12.46)	1	15.4 (14.1–17)	1
	AM-PLGA-NPs	6.9 (7–8.3) ^a,b^	3	4.8 (3–5.4) ^f,g^	4
	5-FU	23.5 (20–26)	5.6	36 (29–36.2)	3.6
HuCCT-1	AM	8.20 (5.5–10.06)	1.1	17 (16.9–20.4)	0.76
	AM-PLGA-NPs	7.60 (5.4–8.4) ^c^	2.3	3.8 (3.0–4.2) ^h^	5.2
	5-FU	24.78 (20–25)	5.3	3.1 (2.9–3.2)	41
OUMS-36T-1F	AM	9.10 (7.1–9.37)	1	12.6 (11.3–13.89)	1
	AM-PLGA-NPs	18.00 (15.4–21) ^d,e^	1	19.5 (16.8–26) ^i^	1
	5-FU	133.00 (98–142)	1	130 (121–251)	1

^a^ Significantly different from free AM with *p*-value = 0.04 (Man–Whitney U test). ^b^ Significantly different from 5-FU with *p*-value = 0.04 (Man–Whitney U test). ^c^ Significantly different from 5-FU with *p*-value = 0.04 (Man–Whitney U test). ^d^ Significantly different from free AM with *p*-value = 0.04 (Man–Whitney U test). ^e^ Significantly different from 5-FU with *p*-value = 0.01 (Man–Whitney U test). ^f^ Significantly different from free AM with *p*-value = 0.04 (Man–Whitney U test). ^g^ Significantly different from 5-FU with *p*-value = 0.04 (Man–Whitney U test). ^h^ Significantly different from free AM with *p*-value = 0.04 (Man–Whitney U test). ^i^ Significantly different from 5-FU with *p*-value = 0.01 (Man–Whitney U test).

## Data Availability

Not applicable.
